# Genital hygiene behaviors in women: a cross-sectional study from Turkey

**DOI:** 10.1590/1806-9282.20241760

**Published:** 2025-06-02

**Authors:** Nazan Karahan, Gizem Aydemir, Emine Tekeli şahin

**Affiliations:** 1 Health Sciences University, Gulhane Faculty of Health Sciences, Department of Midwifery – Ankara, Turkey.; 2 Gaziantep University, Faculty of Health Sciences, Department of Midwifery – Gaziantep, Turkey.

**Keywords:** Hygiene, Genital, Women's Health, Health Behavior

## Abstract

**OBJECTIVE:**

The aim of this cross-sectional study was to identify genital hygiene behaviors and the influencing factors in a sample of women from the Turkish population.

**METHODS:**

Data collection was conducted through an online survey distributed via WhatsApp and social media groups (n=357). The data were collected using the "Genital Hygiene Behaviors Scale." Analyses were conducted using the Mann-Whitney U test, Kruskal-Wallis test, Spearman's correlation, and robust analysis of variance.

**RESULTS:**

The mean age of the women was 31.94±9.7 years; 66.1% were university graduates and 49.6% were married. The mean Genital Hygiene Behaviors Scale total score was 94.93±9.8. genital hygiene behaviors were found to be better among women who were older, who had a higher level of education, were employed, were married, who had received genital hygiene education, and who had a history of genital infections in the past year. Additionally, 38.7% of the women reported abnormal vaginal discharge, and those with abnormal discharge exhibited lower genital hygiene behavior scores. Women who practiced vaginal douching were found to exhibit less adequate genital hygiene behaviors. It was determined that the majority of women with a history of genital infections had not received genital hygiene education and obtained the lowest genital hygiene scores (p<0.05).

**CONCLUSION:**

The findings obtained in the present study indicate that women in socioeconomically disadvantaged groups require more education on this subject and that healthcare professionals should seize every opportunity to provide education.

## INTRODUCTION

Genital hygiene is crucial for maintaining vulvovaginal health and preventing genitourinary infections. The genital area is a region with limited exposure to the external environment and contains numerous skin folds due to its anatomical structure. The skin of the genital area is covered with a thin stratum corneum containing large hair follicles, which facilitates the penetration of microbial and other substances^1^. The anatomical proximity of the urethra, anus, and vagina allows for easier microbial transfer. Under normal conditions, defense mechanisms such as vaginal microbiota and acidity (pH 3.5) protect women from infections^2^. However, the anatomical characteristics combined with inadequate or incorrect genital hygiene behaviors (GHBs) create abnormally moist conditions in the area, alter vaginal pH, and increase susceptibility to vulvar symptoms, such as pain, itching, and burning, as well as infections^3^.

Every year, approximately one million women worldwide are diagnosed with urogenital infections. In Turkey, the prevalence of urogenital infections ranges from 37.1 to 79%^4,5^. Although urogenital infections are typically not life-threatening, they can cause complications that lead to serious health problems and significantly impair women's quality of life^6,7^.

The primary goal of preventing urogenital infections is to reduce etiological factors. Women's urogenital anatomy makes them more susceptible to infections, but appropriate and positive genital hygiene practices can significantly reduce this risk. Appropriate GHBs not only protect women from infections but also promote gender equality, empowering women to engage more actively in social, economic, and political life in alignment with the Sustainable Development Goals^8^. Therefore, it is essential for healthcare professionals to provide education on proper GHBs at every opportunity^9^.

Education and counseling that share accurate, current information help women adopt proper GHBs. Health workers should identify knowledge gaps, address barriers, and consider social, cultural, and geographical contexts^10^. Since GHBs are shaped by regional, cultural, and individual factors^11^ and evolves with modern lifestyles^12^, more studies on women's GHBs are essential. This study was conducted to identify GHBs and the influencing factors among a sample of women from the Turkish population.

## METHODS

The present research is a cross-sectional study. It was conducted in Turkey from May 16 to September 30, 2023, using an online survey distributed via WhatsApp and social media groups.

### Sample size and participants

The sample size was calculated based on an effect size of 0.192 obtained from a reference study^13^, with a 95% power and a 0.05 type I error margin, resulting in a minimum sample size of 353 participants. The sample consisted of 357 women who had internet access, were 18 years of age or older, had not reached menopause, and agreed to participate in the study. Women who were pregnant or suspected of pregnancy, were postmenopausal (surgically or naturally), or who had incomplete survey responses were excluded from the study.

### Data collection tools

Data were collected using the "Genital Hygiene Behaviors Scale" (GHBS). The GHBS was developed by Karahan to assess GHBs among Turkish women. The scale consists of 23 questions categorized into three subscales: "General Hygiene," "Menstrual Hygiene," and "Awareness of Abnormal Findings." It is a self-report scale answered on a 5-point Likert scale. Since the scale is designed for all menstruating women and young girls, it does not include questions on sexual behavior, considering the cultural taboos in Turkey. Scores range from 23 to 115. A higher score indicates good GHBs^14^. In this study, the scale's Cronbach's alpha coefficient was 0.84.

### Data collection

Ethical approval for the study (date: 16.05.2023, decision no. 2023/206) was obtained from the authors’ affiliated institution, and the study was conducted in accordance with the Declaration of Helsinki.

Data were collected through an online Google Forms survey shared via WhatsApp and social media groups. The women received an invitation to participate, including information about the purpose of the research and a link to the survey forms. At the beginning of the survey, the questions confirmed participants’ consent and eligibility for inclusion. The survey was structured in a way that allows only one response per participant, and no identifying information was requested to encourage accurate responses.

### Data analysis

Data were analyzed using a statistical software package. The conformity of the data to normal distribution was determined using the Kolmogorov-Smirnov test. Because the data were not normally distributed, Mann-Whitney U and Kruskal-Wallis tests were used for group comparisons, and Spearman's correlation analysis was used for correlation analysis. Robust analysis of variance (ANOVA) was used to compare GHBS scores among women who reported a genital infection (GI) within the past year, stratified by whether they had received genital hygiene education (GHE). The statistical significance level was set at p<0.05.

## RESULTS

The mean age of the women was 31.94±9.7 years; 66.1% were university graduates, 49.6% were married, and 61.3% were unemployed. Among the participants, 34.2% used daily pads, 28.6% performed vaginal douching, 63% had not received any GHE, and 26.6% reported experiencing a genital tract infection within the past year. According to women's self-reports, those with small amounts of clear, odorless, and colorless discharge were classified as having "normal vaginal discharge," while women with white curd-like, yellow-greenish, grayish, or foul-smelling discharge were classified as having "abnormal vaginal discharge." Accordingly, 38.7% of the women had abnormal discharge. When asked, "What would you do if you thought there was a problem with your vaginal discharge?," 55.2% responded that they would wait for it to resolve on its own. The mean GHBS total score of the women was 94.93±9.8. It was found that women with higher education levels, as well as those who were married and employed, had significantly higher GHBS scores. There was a statistically significant positive correlation between women's age and the subscales of General Hygiene, Menstrual Hygiene, Awareness of Abnormal Findings and the total scale score (p<0.05) (Table 1).

**Table 1:** Distribution of sociodemographic and genital hygiene characteristics of women.

Characteristics		n	%
Age (mean±SD): 31.94±9.7			
GHBS* total score (mean±SD): 94.93±9.9			
General Hygiene subscale (mean±SD): 49.64±5.5			
Menstrual Hygiene subscale (mean±SD): 32.21±4.5			
Awareness of Abnormal Findings subscale (mean±SD): 12.07±3.1			
Education level	Secondary education	66	18.5l
	High school	55	15.4
	University and above	236	66.1
Employment status	Employed	138	38.7
	Unemployed	219	61.3
Income status	Income less than expenses	102	28.6
	Income equal to expenses	198	55.5
	Income more than expenses	57	16.0
Marital status	Married	177	49.6
	Single	180	50.4
Use of daily pads	Yes	122	34.2
	No	235	65.8
Genital hygiene education	Yes	132	37.0
	No	225	63.0
Vaginal douching	Yes	102	28.6
	No	255	71.4
Use of products for vaginal cleansing	None	317	88.8
	Vaginal wet wipes	5	1.4
	Baby wet wipes	9	2.5
	Vaginal wash gel	16	4.5
	Baby oil	10	2.8
Action taken for changes in vaginal discharge	Waits for it to pass	197	55.2
	Visits a doctor	160	44.8
Characteristics of vaginal discharge**	Normal	219	61.3
	Abnormal	138	38.7
History of GTI*** in the last year	Yes	95	26.6
	No	262	73.4

* GHBS: Genital Hygiene Behaviors Scale,

** Normal vaginal discharge was defined as small amounts of clear, odorless, and colorless discharge. Abnormal discharge included white curd-like, yellow-greenish, grayish, or foul-smelling discharge.

*** GTI: genital tract infection. SD: standard deviation.

Women who had received GHE scored higher on the scale than those who had not. Women who practiced vaginal douching scored lower on the total scale and the Menstrual Hygiene subscale. The scale scores of women whose vaginal discharge was classified as abnormal were lower than those whose vaginal discharge was classified as normal, and those who waited for changes in discharge to resolve on their own had lower scores than those who consulted a doctor (p<0.05) (Table 2).

**Table 2:** Comparison of Genital Hygiene Behaviors Scale scores according to sociodemographic characteristics.

	Characteristics	n	General Hygiene subscale	Menstrual Hygiene subscale	Awareness of Abnormal Findings subscale	Scale Total
			Mean rank	Mean rank	Mean rank	Mean rank
Education level	High school below	66	194.36	118.56	174.08	149.37
	High school	55	174.14	173.05	178.26	176.37
	University and above	236	175.84	197.29	180.55	187.90
			H=1.814 p=0.404	H=30.900 **p=0.000**	H=0.215 p=0.898	H=7.241 **p=0.027**
Employment status	Employed	138	185.70	205.16	193.22	201.01
	Unemployed	219	174.78	162.52	170.04	165.13
			z=-0.975 p=0.330	z=-3.812 **p=0.000**	z=-2.111 **p=0.035**	z=-3.202 **p=0.001**
Marital status	Married	177	205.32	189.64	192.11	191.21
	Single	180	153.11	168.18	166.11	166.99
			z=-4.789 **p=0.000**	z=-1.970 **p=0.039**	z=-2.430 **p=0.015**	z=-2.219 **p=0.026**
Previous genital hygiene training	Yes	132	208.08	199.13	207.65	213.96
	No	225	161.94	167.19	162.19	158.49
			z=-4.085 **p=0.000**	z=-2.830 **p=0.005**	z=-4.104 **p=0.000**	z=-4.906 **p=0.000**
Vaginal douching	Yes	102	168.28	161.91	168.74	159.75
	No	255	183.29	185.84	183.10	186.70
			z=-1.243 p=0.214	z=-1.985 **p=0.047**	z=-1.213 p=0.225	z=-2.231 **p=0.026**
Vaginal discharge characteristics	Normal	219	190.52	185.60	182.26	189.17
	Abnormal	138	160.72	168.53	173.83	162.86
			z=-2.662 **p=0.008**	z=-1.526 p=0.127	z=-0.767 p=0.443	z=-2.347 **p=0.019**
Action taken for changes in vaginal discharge	Waits for it to pass	197	153.16	164.00	129.85	141.29
	Visits a doctor	160	210.81	197.47	239.52	225.43
			z=-5.259 **p=0.000**	Z=-3.055 **p=0.002**	Z=-10.199 **p=0.000**	Z=-7.667 **p=0.000**
	Age	**r**	0.242	0.118	0.128	0.116
		**p**	**0.000**	**0.026**	**0.016**	**0.029**

H: Kruskal-Wallis test; z: Mann-Whitney U test, r=Spearman's correlation coefficient. Statistically significant values are denoted in bold.

According to the results of the robust ANOVA, the mean GHBS score for women who had received GHE was 99 compared to 94 for those who had not, showing a statistically significant difference (p=0.001). A statistically significant difference was also found in the mean total scale scores based on whether participants had experienced a GI in the past year (p=0.029). The mean GHBS score was 95.2 among those with no GI history and 98 among those with a GI history. There was no statistically significant difference in mean scores based on the interaction between the GHE and GI history (p=0.781). The mean scores were 98.3 for those who received education and had no infection, 100.4 for those who received education and had an infection, 93.4 for those who did not receive education and had no infection, and 96.1 for those who did not receive education but had an infection (Table 3).

**Table 3:** Comparison of Genital Hygiene Behaviors Scale scores by genital hygiene education status among women with a history of genital infections.

History of genital infection	GHBS* total scores				Test statistic**	p
	Education on genital hygiene		Total			
	Received	Not received				
No	98.3 (0.74)	93.4 (0.78)	95.2 (0.59)	Education	18.5	**0.001**
Yes	100.4 (1.32)	96.1 (1.38)	98.0 (1.04)	GTI	4.972	**0.029**
Total	99.0 (0.72)	94.0 (0.68)		Education×GTI	0.078	0.781

* GHBS: Genital Hygiene Behaviors Scale.

** Robust ANOVA; mean (standard error); GTI: genital tract infection. Statistically significant values are denoted in bold.

## DISCUSSION

It is important for women to have proper GHBs to maintain vulvovaginal health. In this study, the mean GHBS score of the participants was 94.93±9.86. Accordingly, like previous studies in the Turkish population^5,13,15^, it is concluded that women's GHBs are not at the desired level and need to be improved. In this study, GHBS scores increased with age, education level, marriage, and employment. The effects of age and educational level on GHBs have been demonstrated in previous studies^7,13,16^. Improved GHBs with age may stem from increased hygiene awareness due to sexual activity. Society often views women's genitals as requiring constant care to meet norms, idealizing them as odorless and clean^17,18^. Women with higher education levels are more likely to join the workforce, where economic independence enhances access to hygiene products and information^19^. Our findings support this information.

In this study, it was found that 28.6% of the women practiced vaginal douching and had lower GHBs. Vaginal douching disrupts the normal vaginal pH and flora, weakens natural defenses, and fosters an environment that increases susceptibility to infections^20^. Our findings suggest that women who practice vaginal douching lack sufficient knowledge about proper GHBs. Vaginal douching is often practiced by women who are described as vulnerable, with lower levels of education and income, and these women need more health education^20,21^.

The relationship between poor hygiene and abnormal vaginal discharge is well known. In this study, women with abnormal vaginal discharge had lower GHB scores. It was also found that women who noticed a disturbing change in their vaginal discharge waited for it to go away spontaneously instead of going to a doctor (55%) and these women had lower GHBS scores. Similar results were reported in other studies conducted in Turkey^20,21^. We believe that these women avoid gynecological examinations. Some women may find gynecological examinations embarrassing due to emotional or psychological issues^22,23^. A systematic review found that women avoid human papillomavirus screening, which involves a gynecological examination, due to fear, embarrassment, physical limitations, or cultural/religious reasons^24^. Our findings show that women with poor genital hygiene avoid gynecological examinations, have inadequate health-seeking behavior, and are more disadvantaged.

Educational interventions positively impact GHBs. It is crucial for healthcare professionals to seize every opportunity to provide GHE^24^. This study found that women who received GHE had higher GHBS scores, but 63% of the women had not received education. No statistically significant differences were found in GHBS scores based on GHE or a history of GIs. However, it was found that women who had experienced a GI and received GHE (100.4) and those who had not experienced a GI but received GHE (98.3) had higher scores on the scale. Unsurprisingly, women with a history of GI may try to prevent future infections by paying attention to hygiene. Despite having a history of GIs (n=95) and visiting a healthcare professional for treatment, most of these women did not receive GHE and obtained lower scores on the GHBS, indicating a missed opportunity. Women generally do not receive GHE from health personnel^12^. The key implication for clinical practice is that health workers should provide GHE to all women presenting for treatment.

This study provides health professionals with an important perspective on women's GHBs. However, the study has some limitations. These include the facts that the results could not be generalized due to the online nature of the survey, that it did not include information on sexual health history, that the source of GHE was not investigated, and that women with prejudices may have avoided participating due to the special nature of the subject. It is recommended that these limitations be taken into consideration in future studies.

## CONCLUSION

In the present study, it was found that women's GHBs were not at the desired level, and that age, education level, employment status, and marital status were among the factors negatively affecting genital health. GHE is an important factor positively affecting GHBs. One of the most striking findings of our study was the determination that health workers missed the opportunity to provide GHE. Although there were women in our study who had a history of GIs and encountered a healthcare worker for treatment, we found that the majority of these women did not receive GHE. These results highlight missed opportunities for providing GHE to women receiving healthcare and underscore the importance of healthcare professionals seizing every opportunity to deliver such education. By doing so, it may be possible to prevent genitourinary infections and improve women's quality of life.

## ETHICAL APPROVAL

The study was performed in accordance with the ethical standards of the institutional and/or national research committee and with the 1964 Helsinki Declaration and its later amendments and the Good Clinical Practice guidelines. Before starting the study, ethics committee approval numbered 46418926 (2023/206) was obtained from the Gülhane Scientific Research Ethics Committee of the University of Health Sciences. Informed consent was obtained from all individual participants included in the study.

## References

[1] Chen Y, Bruning E, Rubino J, Eder SE (2017). Role of female intimate hygiene in vulvovaginal health: global hygiene practices and product usage. Womens Health (Lond).

[2] Graziottin A (2024). Maintaining vulvar, vaginal and perineal health: clinical considerations. Womens Health (Lond).

[3] Daher A, Albaini O, Siff L, Farah S, Jallad K (2022). Intimate hygiene practices and reproductive tract infections: A systematic review. Gynecol Obstetr Clin Med.

[4] Calik KY, Erkaya R, Ince G, Yıldız NK (2020). Genital hygiene behaviors of women and their effect on vaginal infections. Clin Exp Health Sci.

[5] Cankaya S, Ege E (2014). Evli kadınların genital hijyen davranışlarının ürogenital semptomlar ile ilişkisi. Turkiye Klinikleri J Nurs Sci.

[6] Umami A, Sudalhar S, Lufianti A, Paulik E, Molnár R (2021). Factors associated with genital hygiene behaviors in cervical cancer patients in Surakarta, Indonesia. Nurse Media J Nurs.

[7] Bardin MG, Giraldo PC, Benetti-Pinto CL, Sanches JM, Araujo CC, Amaral RLGD (2022). Habits of genital hygiene and sexual activity among women with bacterial vaginosis and/or vulvovaginal candidiasis. Rev Bras Ginecol Obstet.

[8] United Nation (2016). Sustainable development goals.

[9] Gandhi AB, Madnani N, Thobbi V, Vora P, Seth S, Shah P (2022). Intimate hygiene for women: expert practice points. Int J Reprod Contracept Obstet Gynecol.

[10] Gomes JM, Silva BM, Santos EFS, Kelly PJ, Costa AS, Takiuti AD (2020). Human papillomavirus (HPV) and the quadrivalent HPV vaccine among Brazilian adolescents and parents: factors associated with and divergences in knowledge and acceptance. PLoS One.

[11] Murina PF, Graziottin A, Bagot O, Panay N, Thamkhantho M, Shaw SW (2021). Real-world practices and attitudes towards intimate self- care: results from an international women's survey. J Gynecol Obstet Hum Reprod.

[12] Ruiz C, Giraldo PC, Sanches JM, Reis V, Beghini J, Laguna C (2019). Daily genital cares of female gynecologists: a descriptive study. Rev Assoc Med Bras (1992).

[13] Durmuş MK, Zengin N (2020). Kadınların genital hijyen davranışlarının incelenmesi. J Health Pro Res.

[14] Karahan N (2017). Development of a genital hygiene behavior scale: validity and reliability study. Istanb Med J.

[15] Gözüyeşil E (2020). Investigation of genital hygiene behavior: an example of slum area. Ortadogu Med J.

[16] Soares JM, Oliveira HMC, Luquetti CM, Zuchelo LTS, Arruda Veiga EC, Raimundo JZ (2022). Adolescents’ knowledge of HPV and sexually transmitted infections at public high schools in São Paulo: a cross-sectional study. Clinics (Sao Paulo).

[17] Holdcroft AM, Ireland DJ, Payne MS (2023). The vaginal microbiome in health and disease-what role do common intimate hygiene practices play?. Microorganisms.

[18] Crann SE, Jenkins A, Money DM, O’Doherty KC (2017). Women's genital body work: health, hygiene and beauty practices in the production of idealized female genitalia. Feminism Psychol.

[19] Wireko S, Ofosu M, Agyemang F, Dankluvi HE, Cobbina AE (2024). Vaginal douching and health risks among young women. Health Sci Rep.

[20] Yarıcı F, Mammadov B, Necipoğlu D (2023). Evaluation of vaginal discharge and genital hygiene habits in women: Turkish Republic of North Cyprus Example. Cyprus J Med Sci.

[21] Özcan H, Arık S, Esen UG, Aslan N (2020). Genç kadınların vajinal akıntıyı algılama durumu ve vajinal akıntıya yönelik geleneksel uygulamaları. GUSBD.

[22] Lorenzi NPC, Termini L, Longatto A, Tacla M, Aguiar LM, Beldi MC (2019). Age-related acceptability of vaginal self-sampling in cervical cancer screening at two university hospitals: a pilot cross-sectional study. BMC Public Health.

[23] Braz NS, Lorenzi NP, Sorpreso IC, Aguiar LM, Baracat EC, Soares-Júnior JM (2017). The acceptability of vaginal smear self-collection for screening for cervical cancer: a systematic review. Clinics (Sao Paulo).

[24] Abiç A, Yatmaz G, Altinisik M, Can AA (2024). Effects of planned education on genital hygiene behavior of adolescent females in a secondary school: a quasi-experimental study in Northern Cyprus. Afr J Reprod Health.

